# Plasma zinc levels, anthropometric and socio-demographic characteristics of school children in eastern Nepal

**DOI:** 10.1186/1756-0500-7-18

**Published:** 2014-01-09

**Authors:** Ashwini Kumar Nepal, Basanta Gelal, Kisundeo Mehta, Madhab Lamsal, Paras Kumar Pokharel, Nirmal Baral

**Affiliations:** 1 Department of Biochemistry, Faculty of Medicine, B.P. Koirala Institute of Health Sciences, Dharan, Nepal; 2 Department of Community Medicine, Faculty of Medicine, B.P. Koirala Institute of Health Sciences, Dharan, Nepal

**Keywords:** Plasma zinc levels, Zinc deficiency, Anthropometry, Nepal

## Abstract

**Background:**

Zinc deficiency is a major public health problem in many developing countries including Nepal. The present study was designed to assess the prevalence of zinc deficiency and to study the association of zinc deficiency with anthropometric and socio-demographic variables, in school children of eastern Nepal.

**Methods:**

This cross-sectional study included total 125 school children of age group 6–12 years from Sunsari and Dhankuta districts of eastern Nepal. Plasma zinc level was estimated by Flame Atomic Absorption Spectroscopy.

**Results:**

The Median interquartile range (IQR) values of zinc in the two districts Sunsari and Dhankuta were 5.9 (4.4, 7.9) μmol/L and 5.8 (4.3, 8.4) μmol/L respectively. A total of 55 children (87.3%) in Sunsari and 52 (83.9%) in Dhankuta had zinc deficiency, no significant difference was observed in the Median (IQR) plasma zinc levels (p = 0.9) and zinc deficiency patterns (p = 0.3) of the two districts. Significant differences were observed in the plasma zinc levels (p = 0.02) and zinc deficiency patterns (p = 0.001), of the school children having age groups 6–8 years than in 9–10 and 11–12 years of age, and zinc deficiency patterns between male and female school children (p = 0.04) respectively.

**Conclusions:**

The present study showed higher prevalence of zinc deficiency among school children in eastern Nepal. In our study, zinc deficiency was associated with both sex and age. The findings from the present study will help to populate data for policy implementation regarding consumption and supplementation of zinc.

## Background

Zinc (Zn) deficiency is a major public health problem in many developing countries including Nepal [1,2]. However, its prevalence is still unknown in most populations [2]. Zn deficiency and its adverse effects are associated with poor growth, immune function depression and increased susceptibility to infections [3]. Manifestation of moderate deficiency of Zn includes growth retardation and male hypogonadism in adolescents, cell-mediated immune dysfunction, and abnormal neurosensory changes [4]. In Nepal, very few studies have been conducted so far, regarding Zn deficiency with randomized control trials (RCT). In a RCT conducted by Tielsch et al, 2007 in central Nepal, mean(SD) values of Zn were 11.0 (2.1) μmol/L in placebo and 11.8 (2.4) μmol/L in Zn supplemented children respectively [1]. Supplementation trials of Zn in pneumonia [5,6], and other diseases affecting mortality [1], anthropometry [7,8] and socio-demographic variables [9,10] have been conducted in Nepalese populations in the past decade. Prevalence and the burden of Zn deficiency in the eastern part of Nepal are yet to be explored. Previous studies being mainly focused on pre-school children or infants, there is a paucity of data on the Zn status of the school children of Nepal. These school children form the bulk of the growing children population, being more vulnerable to deficiency of Zn and associated manifestations. Hence, the present study was designed to assess the prevalence of Zn deficiency, and its association with anthropometric and socio-demographic variables, in ethnic Nepalese school children from Sunsari and Dhankuta districts of eastern Nepal.

## Methods

### Study sites and subjects

This cross-sectional study was conducted in a one-year period from August 2009 to August 2010 in school children (n = 125), 6–12 years of age, from two schools each of the two districts Sunsari (plains) and Dhankuta (hills) from eastern Nepal. School children participating in this study had not received any micronutrient supplements of Zn or any tablet forms of Zn. The approximate population of Sunsari and Dhankuta at the time of the study were 700,000 and 200,000 respectively.

### Anthropometric and socio-demographic evaluation

Height was measured at nearest 0.1 cm and weight was adjusted to nearest 0.1 kg. Well-calibrated equipments were used and trained health professionals performed the anthropometric measurements. Stunting (height for age < z scores −2.0 SD), wasting (weight for age z scores < −2.0 SD), thinness (BMI for age z scores < −2.0 SD), underweight (weight for height z scores < −2.0 SD) and overweight (weight for height z scores > 2.0 SD) status were defined as per the World Health Organization (WHO), 2007 and Centers for Disease Control and Prevention (CDC), 2000 criteria for growth charts [10]. Age, sex, and the geographical location (hilly or plain) were recorded as the socio-demographic variables.

### Laboratory procedures

Blood samples (2–3 ml) were collected by venipuncture in EDTA coated vacutainer (BD vacutainer, USA) and were transported to the laboratory maintaining cold chain. The blood samples were centrifuged at 3000 g for 10 mins, and plasma was stored at −20°C until analysis. Plasma was thawed before analysis, and then mixed gently by inverting the tubes. Plasma Zn level was estimated using Flame Atomic Absorption Spectrometry (Thermo Elemental, UK) [11]. All test tubes for Zn analysis were thoroughly acid washed (0.1% Nitric acid) and rinsed with double distilled deionized water. Plasma sample was diluted five times in deionized water, i.e. 500 μL of plasma was diluted with 2 mL deionized water. The instrumental gas flow settings were adjusted and the aspiration rate was established to optimize signal and minimize background noise. Zn working calibrators were aspirated sequentially from the most dilute to the most concentrated, aspiration was continued until the readings were stable. The resulting values were used to establish the calibration curves by use of a least squares regression fit. The specimen concentration was calculated from the absorbance readings by interpolation from the calibration curve. Mean ± SD values for quality control sera for Zn (Seronorm Trace Elements Serum L_1_ and L_2_, Norway) were 28.5 ± 0.8 μmol/L (CV 2.9%, n = 5) for L_1_ and 32.4 ± 2.6 μmol/L (CV 8.1%, n = 5) for L_2_. The corresponding certified values were 26.6 (range 24.1-29.1 μmol/L) for L_1_ and 37.3 (range 27.1-47.5 μmol/L) for L_2_ respectively. Zn deficiency was defined as per the WHO criteria: < 9.9 μmol/L for school-age children of < 10 years of age, < 10.7 μmol/L for male school children of >10 years and < 10.15 μmol/L for female school children of >10 years of age respectively [12].

### Ethical clearance

School children, teachers and guardians were briefly explained about the purpose of the study, with educational information regarding Zn deficiency. Ethical approval and written consent was obtained from the parents or guardians of the school children and the school-teachers, to collect the blood samples for the plasma Zn estimation. The ethical clearance for this study was approved from the Institutional Ethical Review Board of B.P. Koirala Institute of Health Sciences (BPKIHS), Dharan, Nepal.

### Statistical analysis

Data were analyzed with Statistical Package for Social Sciences (SPSS) version 16.0 (SPSS Inc., Chicago, USA). Data were tested for normality using Kolmogorov Smirnov test. Chi Square test was applied to compare the association of qualitative non-parametric data. Man Whitney test and Krushkal Wallis test was applied for the non-parametric numerical data. Binary logistic regression was applied to compare Zn deficiency patterns in male and female school children. Pairwise differences of plasma Zn levels between males Vs females were compared after adjustment for age and vice versa by multiple logistic regression. Anthropometric indices, height for age Z scores, weight for age Z scores, BMI for age Z scores and weight for height Z scores were calculated using Epi Info version 6.0 (CDC, USA) and also manually compared with the WHO growth reference charts, 2007 and CDC Growth Charts, 2000. P values less than 0.05 were considered as statistically significant at 95% confidence intervals (CI).

## Results

### Socio-demographic and anthropometric variables

The Mean ± SD age of school children enrolled for the study (n = 125) were 9.2 ± 1.9 years with Mean ± SD height and weight 12.3 ± 11.2 centimeters and 23.4 ± 6.2 kilograms respectively. Anthropometric and socio-demographic variables including: stunting, wasting, thinness, underweight and overweight status and the distribution of school children in categories of age groups, sex and districts are explained in Table 1.

**Table 1:** Plasma zinc levels and zinc deficiency in various categories

**Variables**	**Categories**	**N (%)**	**Median IQR plasma zinc levels (μmol/L)**	**P value**	**Zinc deficient n (%)**	**Zinc sufficient n (%)**	**P value**
Age (years)	6-8	50(40.0)	6.7(4.8,10.2)	0.02	37(74.0)	13(26.0)	0.001
	9-10	42(33.6)	5.6(4.3,7.1)		37(88.1)	5(11.9)	
	11-12	33(26.4)	5.6(4.0,7.2)		33(100)	0(0)	
Sex	Male	68(54.4)	5.8(4.5,7.7)	0.57	62(91.2)	6(8.8)	0.04
	Female	57(45.6)	5.9(4.3, 8.5)		45(78.9)	12(21.1)	
Districts	Sunsari	63(50.4)	5.9(4.4, 7.9)	0.9	55(87.3)	8(12.7)	0.3
	Dhankuta	62(49.6)	5.8(4.3,8.4)		52(83.9)	10(16.1)	
Stunting	Present	43(34.4)	5.7(4.5,7.4)	0.59	39 (90.7)	4(9.3)	0.1
	Absent	82(65.6)	5.9(4.2,8.6)		68(82.1)	14(17.9)	
Thinness	Present	19(15.2)	5.6(3.8,7.5)	0.22	17(89.5)	2(10.5)	0.4
	Absent	106(84.8)	5.9(4.4,7.9)		90(84.9)	16(15.1)	
Wasting	Present	4(3.2)	5.7(3.7,9.7)	0.78	3(75)	1(25)	0.4
	Absent	121(96.8)	5.9(4.3,7.7)		104(86)	17(14)	
Underweight	Present	37(29.6)	5.6(4.5,7.7)	0.781	29(78.4%)	8(21.6%)	0.5
	Absent	88(70.4%)	5.9(4.3,7.7)		78(88.6%)	10(11.4%)	
Overweight	Present	4(3.2%)	5.0(4.1,6.0)	0.362	4(100%)	-	0.1
	Absent	121(96.8%)	5.9(4.3,7.8)		103(85%)	18(14.9%)	

Man Whitney U test and Krushkal Wallis test was applied to compare the non-parametric numerical data, Chi Square test was applied to associate the non-parametric qualitative data, the level of significance was at 95% CI.

### Plasma zinc levels and its association with socio-demographic factors

Plasma Zn levels were not normally distributed, as tested by Kolmogorov-Smirnov test (n = 125, p value < 0.001). The median interquartile range (IQR) values of plasma Zn levels in Sunsari and Dhankuta were 5.9 (4.4,7.9) μmol/L and 5.8 (4.3,8.4) μmol/L, where as mean ± SD values and median IQR of plasma Zn in overall school children were 6.8 ± 3.5 μmol/L and 5.8 (4.3,7.7) respectively. Zn deficient school children were 55(87.3%) in Sunsari and 52 (83.9%) in Dhankuta. No significant difference was observed in the median (IQR) plasma Zn levels (p = 0.9) and Zn deficiency patterns (p = 0.3) of the two districts. There was significant difference in the plasma Zn levels of school children having 6–8 years of age, than 8–10 and 11–12 years of age (p = 0.02), and Zn deficiency patterns of the three age groups (p = 0.001) (Table 1). Borderline significant Zn deficiency patterns were observed among the male and female groups (p = 0.04) but not in their median (IQR) plasma Zn levels (p = 0.5) (Table 1). Male school children were at 2.75 times risk of Zn deficiency than female students; Odds Ratio 2.75 (0.96-7.8) 95% CI as shown by logistic regression. Multivariate analysis of plasma Zn levels with male vs female adjusted with age showed negative associations with age (β = −0.4, 95% CI −0.7 - -0.07, p = 0.01) (step1) Vs (β = −0.3, 95% CI −0.7 - -0.5, p = 0.02) (step 2) and no significant associations with sex (β = −1.6, 95% CI −0.1 - 0.7, p = 0.4) (step 2) respectively (Table 2).

**Table 2:** Associations of age and sex with plasma zinc level

**Variables**	**Regression coefficient (β)**	**Standard error**	**95% Confidence intervals**	**P value**
Step 1				
Constant	10.5	1.5		
Age	−0.4	0.1	−0.7 - -0.07	0.01
Step 2				
Constant	10.6	1.5		
Age	−0.3	0.1	−0.7 - -0.5	0.02
Sex	−1.6	0.6	−0.1 - 0.7	0.4

Pair-wise differences of zinc levels between males vs females after adjustment for age and vice-versa were assessed in a multivariate model.

### Plasma zinc levels and its association with anthropometric indices

There were no significant differences in Zn deficiency patterns and plasma Zn levels with stunting, wasting, thinness, underweight and overweight status of the school children (Table 1). Zn deficiency was higher in school children having overweight [100% (4/4) Vs 85% (103/121) (p = 0.1)] followed by stunting [90.7% (39/43) Vs 82.1% (68/82) (p = 0.1)], thinness [89.5% (17/19) Vs 84.9% (90/106) (p = 0.4)], wasting [75% (3/4) Vs 86% (104/121) (p = 0.4)] and underweight [78.4% (29/37) Vs 88.6% (78/88) (p = 0.5)] status, than in Zn sufficient children respectively (Table 1). Plasma Zn levels were lower in school children having overweight [5.0 (4.1,6.0) μmol/L Vs 5.9 (4.3,7.8) μmol/L (p = 0.3)], followed by underweight [5.6 (4.5,7.7) μmol/L Vs 5.9 (4.3,7.7) μmol/L (p = 0.7)], thinness [5.6 (3.8,7.5) μmol/L Vs 5.9(4.4,7.9) μmol/L (p = 0.2)], stunting [5.7 (4.5,7.4) μmol/L Vs 5.9 (4.2,8.6) μmol/L (p = 0.5)], and wasting status [5.7 (3.7,9.7) μmol/L Vs 5.9 (4.3,7.7) μmol/L (p = 0.7)] respectively (Table 1).

## Discussion

Present study showed higher prevalence of Zn deficiency and low plasma Zn levels in school children having age group of 9–10 and 11–12 years age, than 6–8 years age. Some investigators have shown tendency for serum Zn to increase with age [13,14], while others showed no change in serum Zn with increasing age [3,15]. Ma et al, 2008 in China showed that proportions of inadequate Zn intakes, were 15.9% and 12.9% in age groups 4–6 years and 7–10 years of age [14]. Increased requirements of Zn in the growing school age children may be due to the requirement of pubertal growth spurt, hormonal influences and co-existing micronutrient deficiencies [16]. Relationships of Zn deficiency, associated with age can be explained on the basis of confounding effects of pubertal status and tanner stage, constraints on growth due to chronic infection and the co-existence of other growth limiting micronutrient deficiencies such as iodine, as described in a previous study [17].

We found higher prevalence of Zn deficiency and low plasma Zn levels in male subjects than in female subjects, which reflects the higher Zn requirements of male than female, which may be due to higher lean body mass and higher growth rate of male than female. This is supported by Hotz and Brown (2004), who showed male had 2 fold greater risk of being Zn deficient [12]. However, Fesharakinia et al (2009) in East Iran showed the mean Zn levels in serum were markedly higher in girls than in boys [3]. A similar sex related trend in serum Zn concentration, as our study has been documented by some [3,18-20] but not all investigators [13,15,21,22].

In our study, highest prevalence of Zn deficiency was observed in the overweight and the stunted school children, where as lowest plasma Zn levels were found in the school children having overweight and underweight status. Despite the biochemical evidence of association of Zn deficiency with anthropometric status, we did not detect any significant relationships between plasma Zn levels and anthropometric status in our study. Which may be accountable to the relationships associated with the pubertal status of the school children, constraints on growth due to chronic infection and the co-existence of other growth limiting micronutrient deficiencies in addition to Zn [12,17]. The association between serum Zn level and anthropometric status is not so clear, several investigators have found varying results. Singla et al, (1996) reported significant association of height for age z-scores with Zn deficiency among 58 children (3 months-5 years of age) in India [23]. Similarly, association of zinc deficiency with stunting was shown by Fesharakinia et al, 2009 in Iran, where 35% of school children (9–11 years age) who were stunted had zinc deficiency [3]. In a study conducted by Qin et al, 2009 in China among 2400 school children (6–9 years of age) no significant association was found between serum Zn and height for age Z scores (stunting), which was supported by our study [20]. Brown et al, 2002 in a meta-analysis study, concluded that children supplemented with Zn had greater growth increments than control groups and there was no clear pattern of response with change for weight for height Z scores with Zn supplementation [24].

Previous studies in other regions have shown variable plasma Zn levels in school children. Hettiarachchi et al, (2006) reported Zn deficiency in 51.5% and 58.5% boys and girls in a study population of 945 school children (12–16 years) in Srilanka [25]. Some of the previous studies of mean ± SD plasma Zn reported in other parts of the world were: i) Mahmmodi et al, 2001; Iran, age (years) 12–14 (n = 881) 14.6 ± 2.7 μmol/L [26], ii) Arvinitidou et al, 2007; Greece, age (years) 3–14 (n = 105) 15 μmol/L [22], iii) Ohtake et al, 1976; Japan, age (years) 6–12 (n = 156), 14.1 ± 1.9 μmol/L [15], iv) Ndeezi et al, 2009; Uganda, age (years) 1–6 (n = 247) 10 ± 2.9 μmol/L [27] and, v) Amare et al, 2012; Ethiopia, age (years) 10–14 (n = 100), 13.22 ± 6.5 μmol/L [28]. Our study showed mean ± SD plasma Zn (6.8 ± 3.5 μmol/L), which was lower as compared to other studies.

### Limitations

The present study has few limitations. Firstly: the key determinants of Zn status, including: hair zinc levels, iron or hemoglobin status for anemia, presence of acute illness, inflammation markers, including C Reactive Protein and serum Albumin were not estimated in this study. Secondly, the pubertal status and tanner stage of the school children were not known which limits to draw conclusions about effects of zinc deficiency in anthropometric status, and small sample size of our study limits to investigate the prevalence of Zn deficiency in these regions.

## Conclusion

Our study showed considerably low plasma Zn levels in the school children, as compared to other studies. It is recommended that a regular supplementation of Zn should be provided, to Zn deficient groups, in order to prevent the adverse consequences of Zn deficiency. Populations based larger studies should be conducted in these regions to investigate the prevalence of Zn deficiencies and establish the confounding factors of Zn deficiency, such as dietary source, water sources, environmental and genetic factors.

The present study suggested higher prevalence of Zn deficiency in school children of eastern Nepal. In our study, Zn deficiency was associated with both sex and age. The findings from the present study will help to populate data for the implementation of policy regarding consumption and supplementation of Zn, in Zn deficient school children. Also, our study urges the need of educational programs regarding Zn deficiency, micronutrient National surveys in school children including Zn, and supplementation of Zn to the vulnerable groups. The policy-making and concerned governing bodies should work on developing and implementing the policy that Zn should not only be supplemented in pneumonia, diarrhea and other disease conditions, but also careful examination of anthropometric and nutritional indices should be considered. Supplementation to severely malnourished children is also essential for elimination of Zn deficiency. Establishment of normative values of Zn in Nepalese children should also be continued. Further substantial studies should be conducted both by the Government and Non-government organizations to establish the determinants of severe Zn deficiency in these regions.

## Competing interests

The authors declare that they have no competing interests.

## Authors’ contributions

AKN contributed to the study design, acquisition of data, laboratory analysis, analysis and interpretation of data, and drafted the manuscript. BG and KDM contributed to the study design, acquisition of data. ML contributed to study design, interpretation of data, and drafted the manuscript. NB and PKP contributed to the revision of the manuscript. NB contributed to study design. All authors read and approved the final manuscript.
